# Supplementary material for: Lipidome atlas of the adult human brain
Source: Nat Commun. 2024 May 25;15:4455. doi: 10.1038/s41467-024-48734-y (PMC11127996; doi:10.1038/s41467-024-48734-y)
Supplement: Supplementary file 1 — Supplementary Information [file 41467_2024_48734_MOESM1_ESM.pdf]

Supplementary Information

Lipidome atlas of the adult human brain

Maria Osetrova[1], Anna Tkachev[1], Waltraud Mair[1], Patricia Guijarro Larraz[1], Olga Efimova[1], Ilia Kurochkin[1], Elena Stekolshchikova[1], Nickolay Anikanov[1], Juat Chin Foo[2], Amaury Cazenave-Gassiot[2], Aleksandra Mitina[1], Polina Ogurtsova[1], Song Guo[1], Daria M. Potashnikova[3], Alexander A. Gulin[4], Alexander A. Vasin[4,5], Anastasia Sarycheva[1], Gleb Vladimirov[1], Maria Fedorova[6], Yury Kostyukevich[1], Evgeny Nikolaev[1], Markus R. Wenk[2,*], Ekaterina E. Khrameeva[1,*], Philipp Khaitovich[1,*]

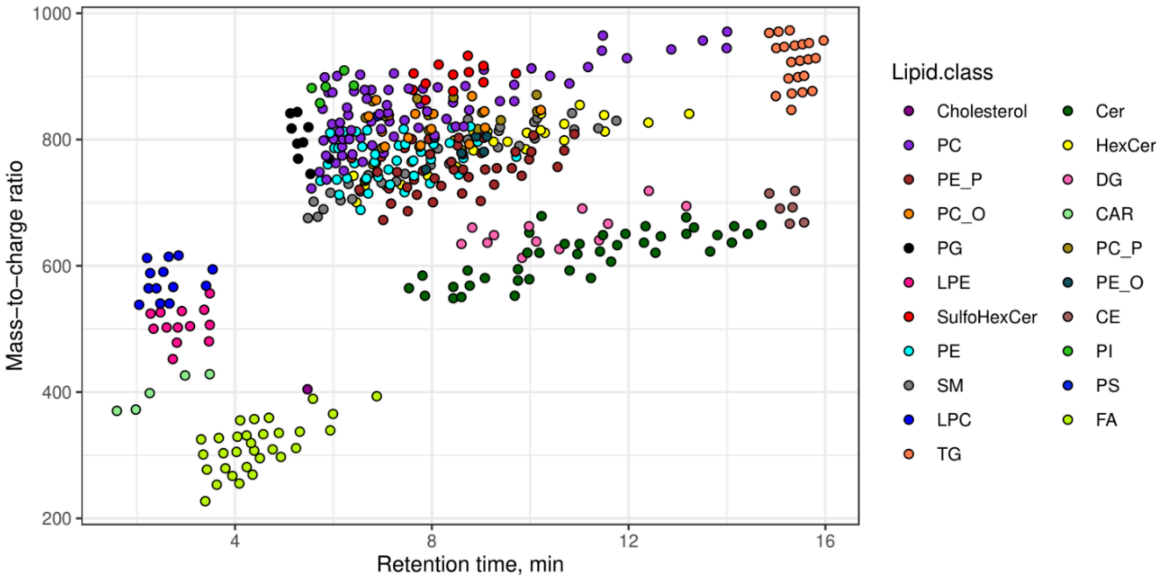

Supplementary Fig. 1. Distribution of 419 annotated HRMS lipids.

Chart displaying the distribution of 419 annotated HRMS lipids based on their mass spectrometric parameters: retention time and mass-to-charge ratio. Each circle represents an annotated lipid compound. Colors represent lipid class annotation. Source data are provided as a Source Data file.

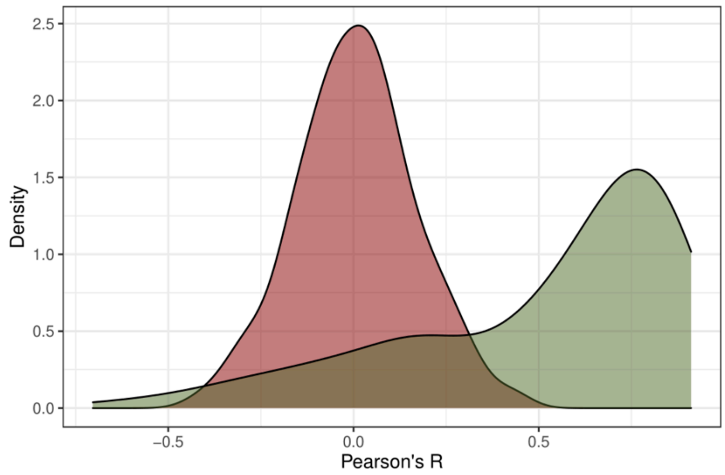

Supplementary Fig. 2. Distribution of the correlation coefficient values between HRMS and MRM measurements of macaque samples.

Distribution of the correlation coefficient values based on the comparison of 156 lipid intensity profiles between HRMS and MRM measurements of macaque samples (*n*=4 animals) (green). Random pairs distribution (red) represent the correlation coefficients calculated between lipid intensity profiles of two macaque datasets with permuted region labels. Source data are provided as a Source Data file.

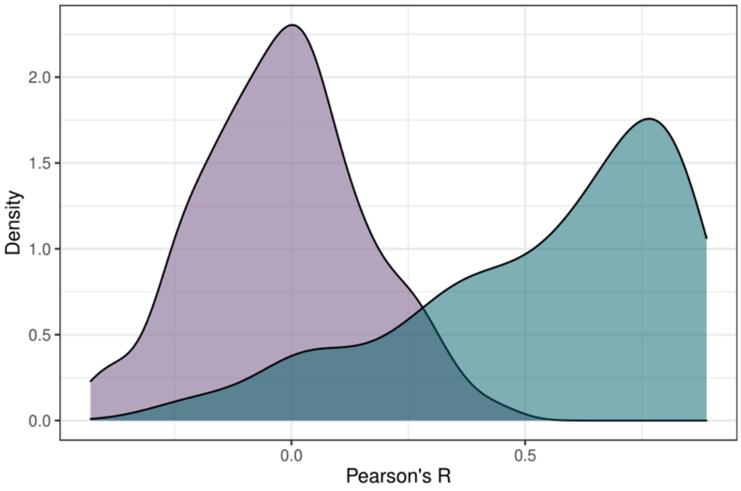

Supplementary Fig. 3. Distribution of the correlation coefficient values between human and macaque MRM measurements.

Distribution of the correlation coefficient values based on the comparison of 216 lipid intensity profiles between human (*n*=4 individuals) and macaque (*n*=3 animals) MRM measurements (marine blue). Random pairs distribution (purple) represent the correlation coefficients calculated between lipid intensity profiles of two MRM datasets with permuted region labels. Source data are provided as a Source Data file.

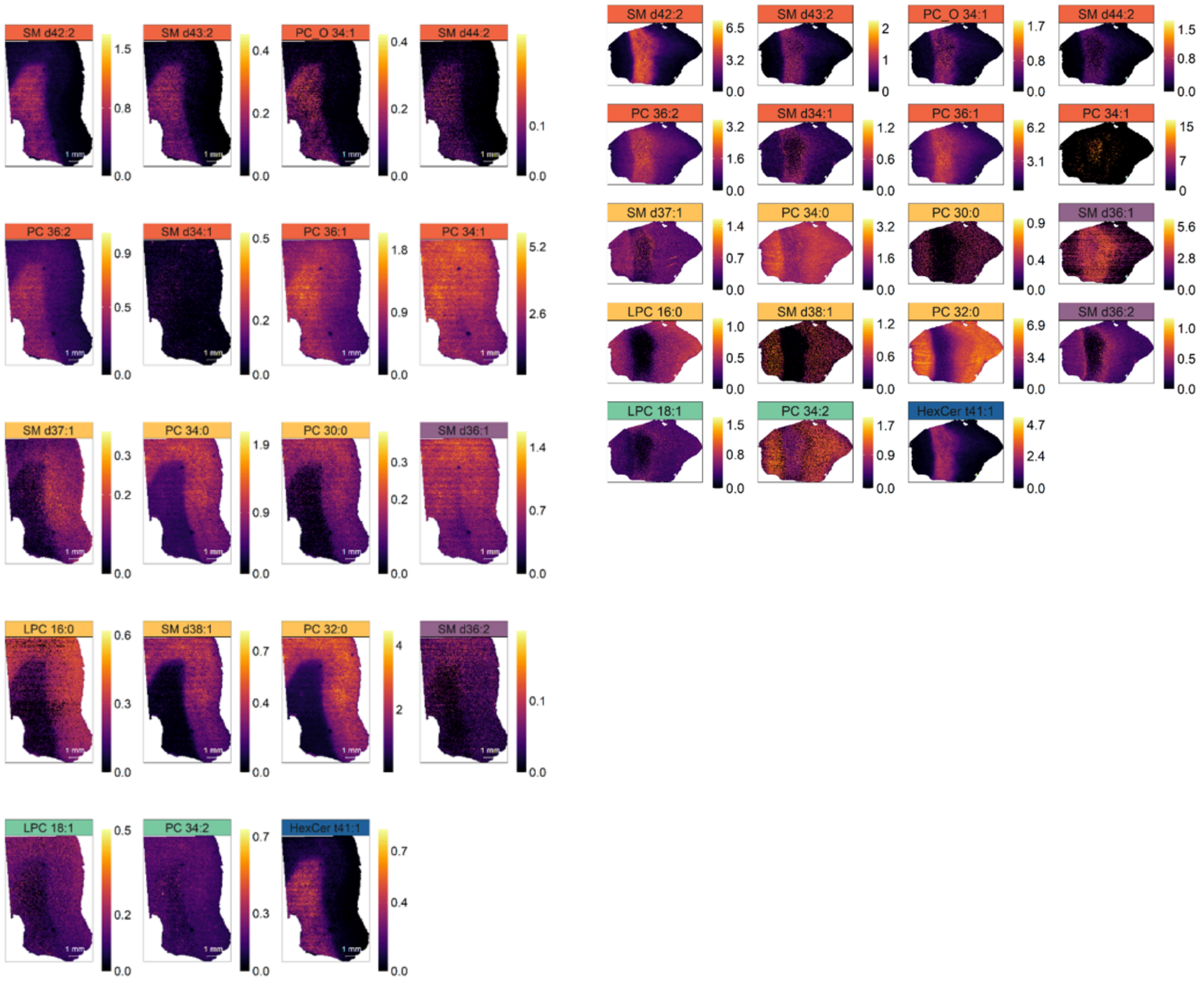

Supplementary Fig. 4. MALDI imaging of human prefrontal cortical sections.

Visualization of the spatial intensity distributions of 19 HRMS-detected lipids using MALDI imaging of human prefrontal cortical sections in two individuals: HC2 (left) and HC3 (right) – individual labels according to Supplementary Table 2. The lipid compound annotation and category assignment are indicated by the label and the color of the bar placed on top of each ion plot, respectively.

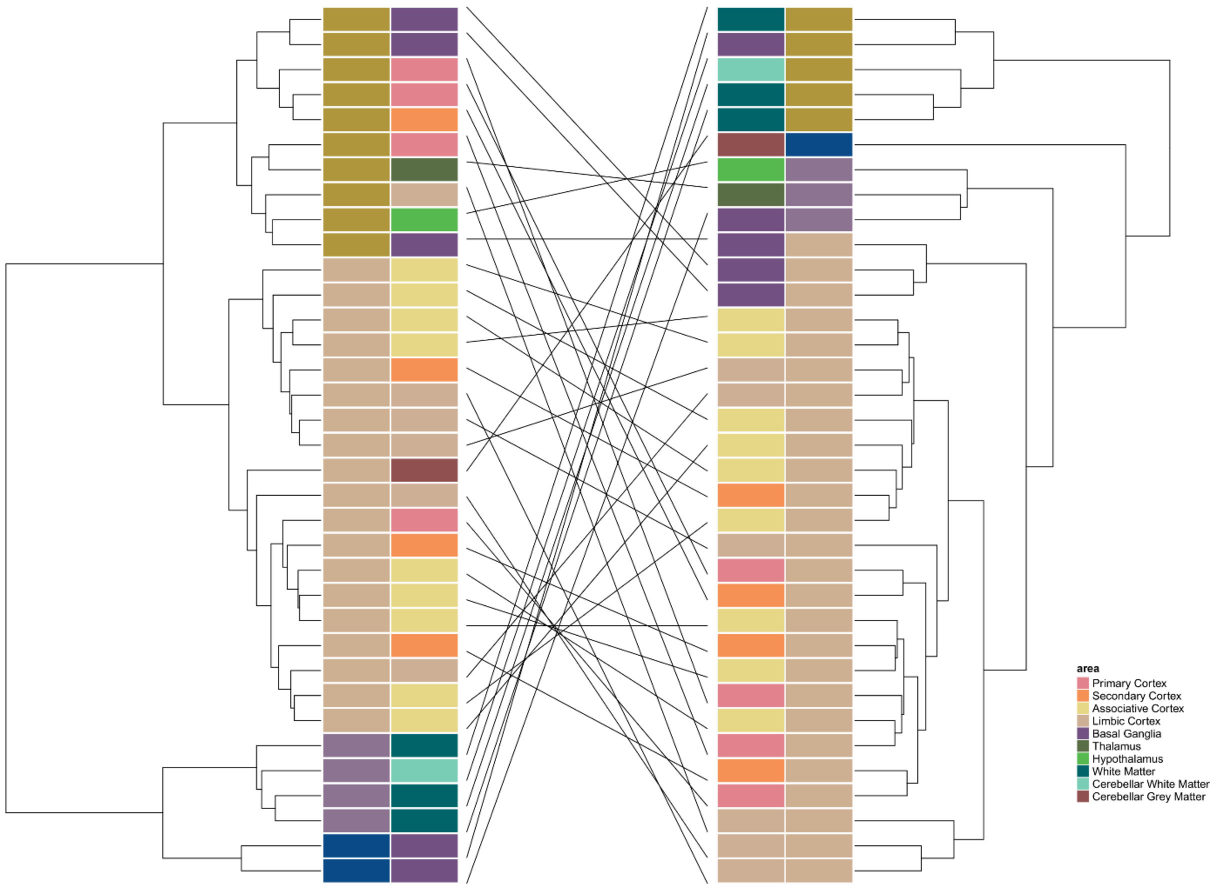

Supplementary Fig. 5. Comparison between lipid and mRNA expression measurements.

Back-to-back comparison of hierarchical clustering dendrograms constructed based on lipid (left) and mRNA expression (right) measurements derived from the same tissue samples representing 35 brain regions in adult human individuals (*n*=4 individuals). Dendrogram leaf colors indicate suggested clusters (pale colors) and anatomical structures (bright colors). Source data are provided as a Source Data file.

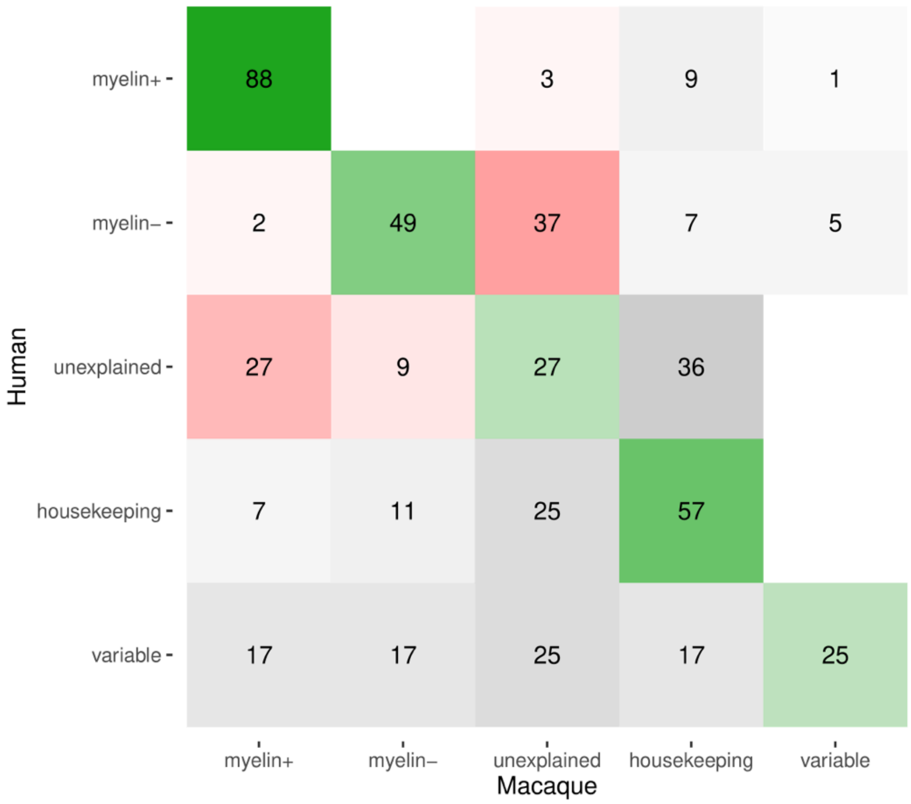

Supplementary Fig. 6. Correspondence between human and macaque MRM data.

Correspondence (in %) of the lipid placement into the five categories between human (*n*=4 individuals) and macaque (*n*=3 animals) MRM data. Green color indicates perfect match, red – mismatch, and gray – non-contradictory alternative assignment. Color intensity reflects the number of overlapping lipids.

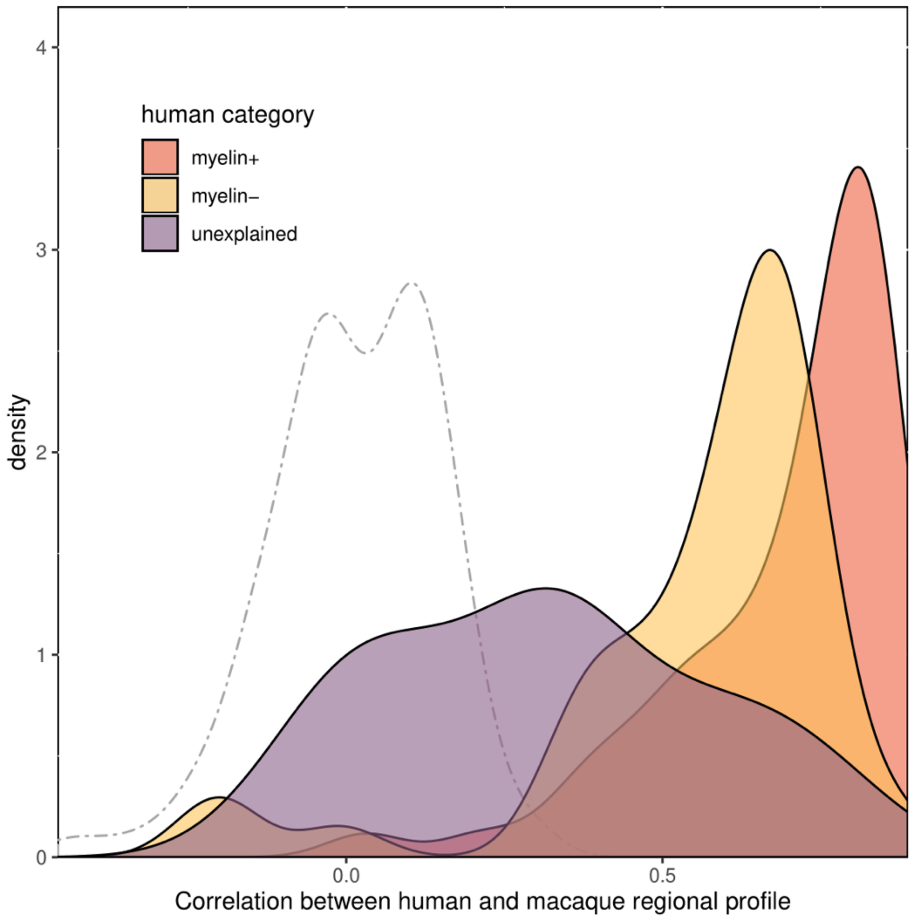

Supplementary Fig. 7. Distribution of the correlation coefficient values between human (*n*=4 individuals) and macaque (*n*=3 animals) MRM measurements.

The distribution is based on the comparison of intensity profiles of lipids contained in the three main categories according to human data-based classification. Number of lipids in analysis: myelin^{+} N=113, myelin^{-} N=41, unexplained N=22). Dashed line indicates the random pairs distribution based on the correlation coefficients calculated between lipid intensity profiles of two MRM datasets with permuted region labels. Source data are provided as a Source Data file.

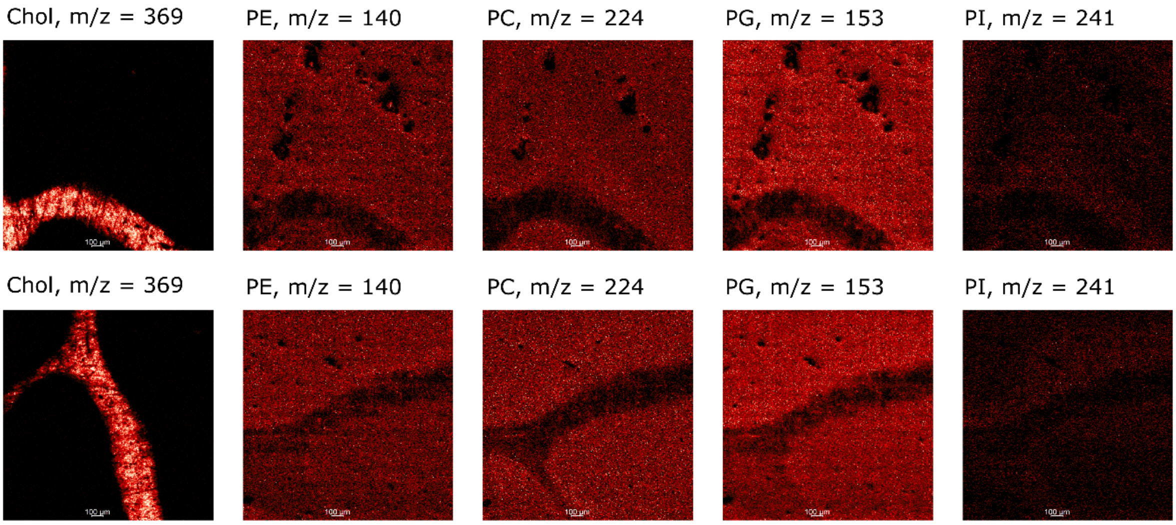

Supplementary Fig. 8. SIMS imaging of human cerebellar sections.

Visualization of the spatial intensity distributions of five lipid head group ions, marked on top of the panels, conducted using SIMS imaging of human cerebellar sections in two individuals: HC1 (upper row) and HC2 (bottom row) – individual labels according to Supplementary Table 2.

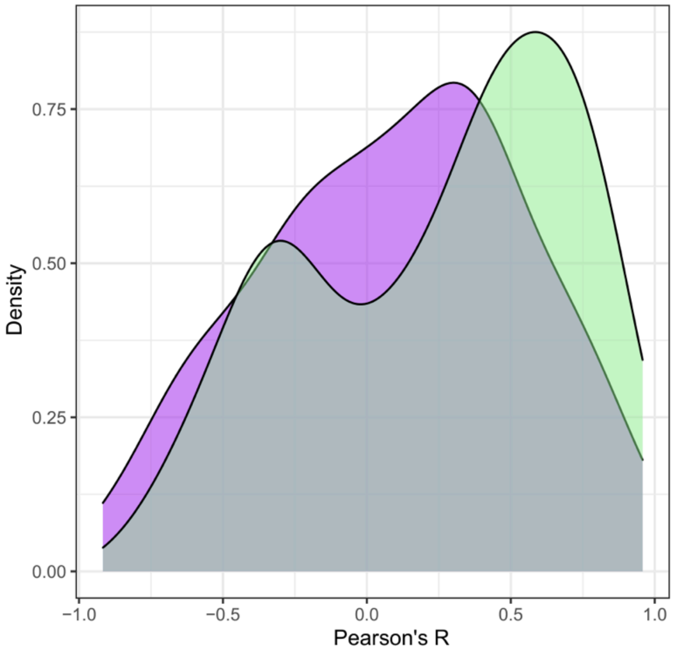

Supplementary Fig. 9. Distribution of the correlation coefficient values between human and mouse HRMS measurements.

Distribution of the correlation coefficient values based on the comparison of 329 lipid intensity profiles between human (n=4 individuals) and mouse (n=3 animals) HRMS measurements (green). Random pairs distribution (purple) represent the correlation coefficients calculated between lipid intensity profiles of two datasets with permuted region labels. The two distributions are significantly distinct (one-sided Wilcoxon signed-rank test, p = 0.00001). Source data are provided as a Source Data file.

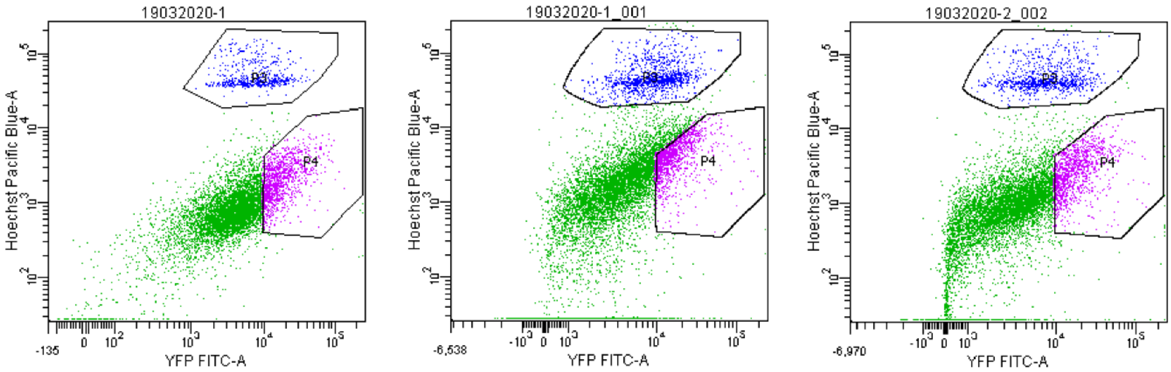

Supplementary Fig. 10. Dot plots of cell populations in the FACS experiment.

Fenced area P4 (purple) represents the population of interest corresponding to the fluorescently labeled fraction enriched in pyramidal neurons' dendrites. Fenced area P3 (blue) represents cell fraction used as a background in the lipidome analysis.

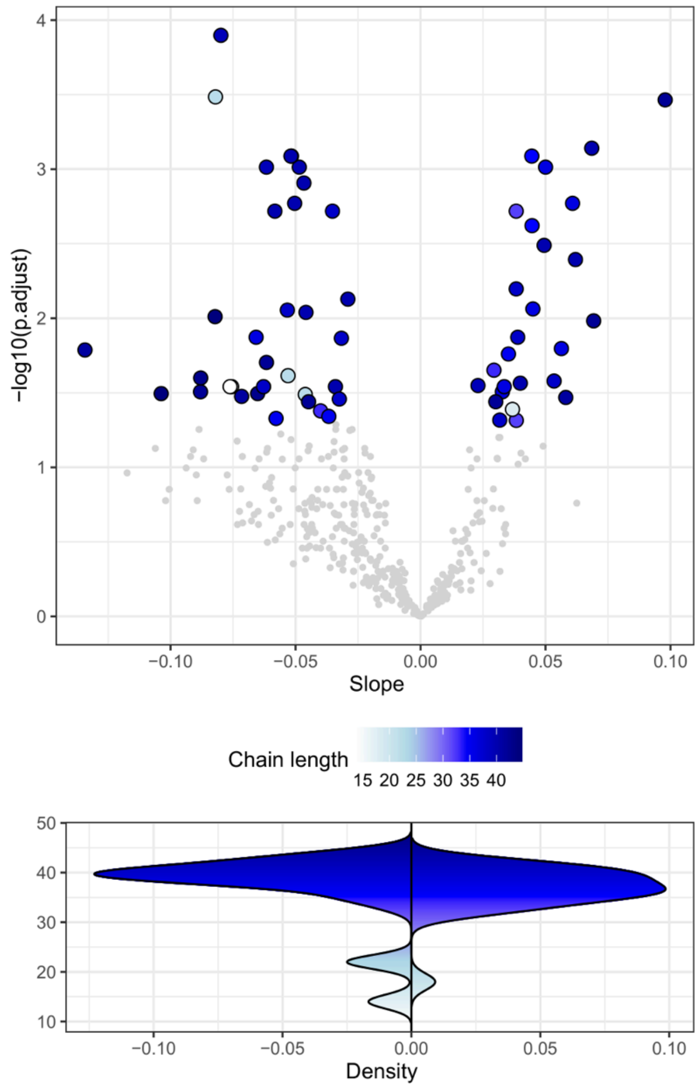

Supplementary Fig. 11. Chain lengths of fatty acid residues in the human brain (*n*=4 individuals).

Volcano (top) and distribution (bottom) plots showing chain lengths of fatty acid residues contained in lipids significantly negatively and positively correlated with HR. There are no significant differences between negatively and positively correlated lipids. Source data are provided as a Source Data file.

Supplementary Table 1. Brain donors' information.

Brain ID	Species	Sex	Age
MA	Macaca mulatta	M	8.5
MB	Macaca mulatta	M	10
MC	Macaca mulatta	F	11
HA	Homo sapiens	M	62
HB	Homo sapiens	F	61
HC	Homo sapiens	F	34
HD	Homo sapiens	M	34

Supplementary Table 2. Brain donors' information for samples used in mass spectrometry imaging experiments (MALDI and ToF-SIMS). Of the four donors, three were involved in SIMS measurements.

Brain ID	Sex	Age, years	PMI, hours	Cause of Death	MALDI, BA22p	ToF-SIMS, Cerebellum
HC1	m	59	24	hypovolemic shock	✓	
HC2	f	62	30	peritonitis	✓	✓
HC3	f	36	30	hepatocellular failure	✓	✓
HC4	f	63	20	acute respiratory failure	✓	✓
