# Supplementary material for: Lipidome atlas of the adult human brain
Source: Nat Commun. 2024 May 25;15:4455. doi: 10.1038/s41467-024-48734-y (PMC11127996; doi:10.1038/s41467-024-48734-y)
Supplement: Supplementary file 3 — Description of Additional Supplementary Information [file 41467_2024_48734_MOESM3_ESM.pdf]

Description of Additional Supplementary Materials

Supplementary Data 1. Brain region information including: anatomical information and processing hierarchy level, human and macaque individuals sampled for these regions, and sMRI values extracted from publications.

Supplementary Data 2. Annotated brain lipids used in the main analysis. (A) Annotated HRMS human brain lipids used in the main analysis. For Hierarchy P-value, the linear model was used, BH-corrected. For correlation to FC P-value, Pearson's R p-value was used, non-corrected. (B) HRMS human tissue sample ID. (C) Normalized HRMS human lipid intensities (for Peak ID information see Supplementary Data 2A). (D) Annotated HRMS macaque brain lipids used in the main analysis. The last column indicates the corresponding human peak ID listed in Supplementary Data 2A. (E) HRMS macaque tissue sample ID. (F) Normalized HRMS macaque lipid intensities (for Peak ID information see Supplementary Data 2D). (G) MRM lipids measured in humans and macaques. The last column indicates human HRMS peak ID (see Supplementary Data 2A). (H) MRM human tissue sample ID. (I) Normalized MRM human lipid intensities (for Peak ID information see Supplementary Data 2G). (J) MRM macaque tissue sample ID. (K) Normalized MRM macaque lipid intensities (for Peak ID information see Supplementary Data 2G).

Supplementary Data 3. Functional analysis of genes associated with lipid categories and the brain processing hierarchy. (A) Gene Onthology (GO) term enrichment in genes associated with lipid categories. Hypergeometric enrichment p-value was calculated, one-sided, BH-corrected. (B) Gene Onthology (GO) term enrichment in genes associated with the brain processing hierarchy. Hypergeometric enrichment p-value was calculated, one-sided, BH-corrected.
