# Supplementary material for: Lipidome atlas of the adult human brain
Source: Nat Commun. 2024 May 25;15:4455. doi: 10.1038/s41467-024-48734-y (PMC11127996; doi:10.1038/s41467-024-48734-y)
Supplement: Supplementary file 7 — Reporting Summary [file 41467_2024_48734_MOESM7_ESM.pdf]

nature portfolio

Corresponding author(s): Philipp Khaitovich

Last updated by author(s): Apr 02, 2024

Reporting Summary

Nature Portfolio wishes to improve the reproducibility of the work that we publish. This form provides structure for consistency and transparency in reporting. For further information on Nature Portfolio policies, see our Editorial Policies and the Editorial Policy Checklist.

Statistics

For all statistical analyses, confirm that the following items are present in the figure legend, table legend, main text, or Methods section.

n/a	Confirmed	
☐	☒	The exact sample size (*n*) for each experimental group/condition, given as a discrete number and unit of measurement
☐	☒	A statement on whether measurements were taken from distinct samples or whether the same sample was measured repeatedly
☐	☒	The statistical test(s) used AND whether they are one- or two-sided<br>*Only common tests should be described solely by name; describe more complex techniques in the Methods section.*
☒	☐	A description of all covariates tested
☐	☒	A description of any assumptions or corrections, such as tests of normality and adjustment for multiple comparisons
☐	☒	A full description of the statistical parameters including central tendency (e.g. means) or other basic estimates (e.g. regression coefficient) AND variation (e.g. standard deviation) or associated estimates of uncertainty (e.g. confidence intervals)
☐	☒	For null hypothesis testing, the test statistic (e.g. *F*, *t*, *r*) with confidence intervals, effect sizes, degrees of freedom and *P* value noted<br>*Give P values as exact values whenever suitable.*
☒	☐	For Bayesian analysis, information on the choice of priors and Markov chain Monte Carlo settings
☒	☐	For hierarchical and complex designs, identification of the appropriate level for tests and full reporting of outcomes
☐	☒	Estimates of effect sizes (e.g. Cohen's *d*, Pearson's *r*), indicating how they were calculated

Our web collection on statistics for biologists contains articles on many of the points above.

Software and code

Policy information about availability of computer code

Data collection	High resolution mass spectrometry data was acquired using Bruker Impact II QTOF with Waters Acquity UPLC System.<br>Targeted MS measurements (MRM) were acquired using Agilent 6495 Triple Quadrupole mass spectrometer coupled with an Agilent 1290 Infinity System.<br>RNA libraries were sequenced on the Illumina HiSeq 4000 system using the 150-bp paired-end sequencing protocol.<br>Cell sorting experiment results were collected using FACSAria SORP instrument (BD Biosciences).<br>Raw MALDI images were collected using Orbitrap tune software, coordinate files obtained using Injector Software.<br>ToF-SIMS measurements were performed using a ToF-SIMS 5 instrument (ION-TOF Gmbh, Germany).
Data analysis	Bruker DataAnalyst Version 4.3 was used for Bruker .d raw data calibration and conversion to .mzXML format. Obtained .mzXML files were then transferred to XCMS software using the xcms package for R version 3.8.2. Further analysis was conducted using custom R scripts.<br>All MRM transitions were integrated with MassHunter Quantitative Analysis software version B.08. Further analysis was conducted using custom R scripts.<br>Raw RNA data was quality assessed, trimmed and mapped onto genomes using fastQC, trimmomatic and hisat2 tools, respectively. Further analysis was conducted using custom R scripts.<br>MRI data was extracted using oro.nifti R package.<br>MALDI ion images were generated from raw files and coordinate files by Image Insight software from Spectroglyph LLC. MALDI raw mass spectra were converted to *.ibd and *.imzML formats using Spectroglyph software. All further processing was done using Cardinal 2.8.0, an R package designed for mass spectrometry imaging data analysis, or custom R scripts.<br>All custom code used in this manuscript is publicly available at https://doi.org/10.5281/zenodo.10908108.

For manuscripts utilizing custom algorithms or software that are central to the research but not yet described in published literature, software must be made available to editors and reviewers. We strongly encourage code deposition in a community repository (e.g. GitHub). See the Nature Portfolio guidelines for submitting code & software for further information.

Data

Policy information about availability of data

All manuscripts must include a data availability statement. This statement should provide the following information, where applicable:

- Accession codes, unique identifiers, or web links for publicly available datasets
- A description of any restrictions on data availability
- For clinical datasets or third party data, please ensure that the statement adheres to our policy

The raw and processed RNA-seq data generated in this study for two brain regions have been deposited in the GEO database under accession number GSE262948 [https://www.ncbi.nlm.nih.gov/geo/query/acc.cgi?acc=GSE262948]. The RNA-data data used in this study for the remaining 33 brain regions are available under accession number GSE127898 [https://www.ncbi.nlm.nih.gov/geo/query/acc.cgi?acc=GSE127898]. All other data generated in this study are provided in the Supplementary Information and Source Data file. Source data are provided with this paper.

Research involving human participants, their data, or biological material

Policy information about studies with human participants or human data. See also policy information about sex, gender (identity/presentation), and sexual orientation and race, ethnicity and racism.

Reporting on sex and gender	Sex was determined based on mRNA expression of Y chromosome genes, gender was not considered, since all samples were postmortem.
Reporting on race, ethnicity, or other socially relevant groupings	We did not consider socially constructed or socially relevant categorization variables in our study.
Population characteristics	All studied invididuals were adults (age range: 34-62 years), included representatives of both sexes (50% females), and represented the same population (East Asians). All human subjects were cognitively healthy and did not have any brain-related diagnoses. All human subjects suffered sudden death with no prolonged agony state from causes not related to brain function.
Recruitment	All human subjects were defined as healthy with respect to the sampled brain tissue by medical pathologists. Potential self-selection bias is unlikely to affect the biochemical characteristics of the brain studied in this work.
Ethics oversight	Informed consent for the use of human brain tissues for research was obtained from all donors or their next of kin by the tissue provider bank. The protocol was approved by the Skoltech Institutional Review Board.

Note that full information on the approval of the study protocol must also be provided in the manuscript.

Field-specific reporting

Please select the one below that is the best fit for your research. If you are not sure, read the appropriate sections before making your selection.

☒ Life sciences ☐ Behavioural & social sciences ☐ Ecological, evolutionary & environmental sciences

For a reference copy of the document with all sections, see nature.com/documents/nr-reporting-summary-flat.pdf

Life sciences study design

All studies must disclose on these points even when the disclosure is negative.

Sample size	We performed an exploratory study and the sample size was determined based on the published studies constructing the human brain transcriptome maps as the closest avaliable analog (Khrameeva et al., 2020, https://doi.org/10.1101/gr.256958.119). We measured the lipidome composition in 75 distinct brain regions dissected from four humans and 38 brain three macaques, which totals to >400 samples, by two mass spectrometry-based techniques (HRMS and MRM). Because each measurement takes >30 min (30 min x 400 samples = 200 hours), excluding the time for the sample preparation and lipid extraction, analyzing a greater number of samples would not be feasible, and running measurement on different machines in parallel would create batch effects. Therefore, the samples sizes were chosen as maximum possible ones for the analysis withing reasonable timeframes.
Data exclusions	No data was excluded from the analysis.
Replication	The human lipidome data was produced from 4 biological replicates. We measured reproducibility by performing principal component analysis (PCA). PCA demonstrated a clear separation by brain regions rather than the replicates. In addition, the results obtained on this main dataset were replicated in a series of independent follow-up experiments. In particular, macaque brain samples were used to replicate the results obtained from human brain data as macaques were raised in a standardized environment, followed by rapid and controlled tissue collection, thus providing a controlled reference for the human brain lipidome quality evaluation. The macaque lipidome data was produced from 3 biological replicates. Additionally, two independent mass-spectrometry techniques (HRMS and MRM) were used to replicate both human and macaque results. We independently measured lipid composition by HRMS and MRM, in 4 human biological replicates and 3 macaque biological replicates.

Randomization	Randomization was applied on the the stages of sample preparation as well as mass spectometric measurements.
Blinding	Blinding was not applicable since only one homogenous group was investigated.

Reporting for specific materials, systems and methods

We require information from authors about some types of materials, experimental systems and methods used in many studies. Here, indicate whether each material, system or method listed is relevant to your study. If you are not sure if a list item applies to your research, read the appropriate section before selecting a response.

Materials & experimental systems

n/a	Involved in the study
☒	☐ Antibodies
☒	☐ Eukaryotic cell lines
☒	☐ Palaeontology and archaeology
☐	☒ Animals and other organisms
☒	☐ Clinical data
☒	☐ Dual use research of concern
☒	☐ Plants

Methods

n/a	Involved in the study
☒	☐ ChIP-seq
☐	☒ Flow cytometry
☒	☐ MRI-based neuroimaging

Animals and other research organisms

Policy information about studies involving animals; ARRIVE guidelines recommended for reporting animal research, and Sex and Gender in Research

Laboratory animals	Adult Tg(Thy1-COP4/EYFP)9Gfng (Thy1-ChR2-YFP) transgenic mice (4-5 months).
Wild animals	No wild animals were used in the study.
Reporting on sex	The Thy1-ChR2-YFP female mice were used in the cell sorting experiment. Sex was assigned by visual inspection. Thy1-ChR2-YFP expression has no sexual difference according to the description of strain by a provider.
Field-collected samples	No field collected samples were used in the study.
Ethics oversight	Adult mice were anesthetized and decapitated following the standard, ethically acceptable procedure. The protocol was approved by the Skoltech Institutional Review Board in accordance with the guidelines on the ethical use of animals. All possible efforts were made to minimize animal suffering, and to reduce the number of animals used per condition by calculating the necessary sample size before performing the experiments.

Note that full information on the approval of the study protocol must also be provided in the manuscript.

Plants

Seed stocks	*Report on the source of all seed stocks or other plant material used. If applicable, state the seed stock centre and catalogue number. If plant specimens were collected from the field, describe the collection location, date and sampling procedures.*
Novel plant genotypes	*Describe the methods by which all novel plant genotypes were produced. This includes those generated by transgenic approaches, gene editing, chemical/radiation-based mutagenesis and hybridization. For transgenic lines, describe the transformation method, the number of independent lines analyzed and the generation upon which experiments were performed. For gene-edited lines, describe the editor used, the endogenous sequence targeted for editing, the targeting guide RNA sequence (if applicable) and how the editor was applied.*
Authentication	*Describe any authentication procedures for each seed stock used or novel genotype generated. Describe any experiments used to assess the effect of a mutation and, where applicable, how potential secondary effects (e.g. second site T-DNA insertions, mosiacism, off-target gene editing) were examined.*

Flow Cytometry

Plots

Confirm that:

☒ The axis labels state the marker and fluorochrome used (e.g. CD4-FITC).

☒ The axis scales are clearly visible. Include numbers along axes only for bottom left plot of group (a 'group' is an analysis of identical markers).

☒ All plots are contour plots with outliers or pseudocolor plots.

☐ A numerical value for number of cells or percentage (with statistics) is provided.

Methodology

Sample preparation	Intact brains were isolated from the sacrificed animals within 3 min postmortem, minced by two cold razor blades on ice cold glass plate and placed in an ice-cold solution of zinc fixative (0.1 M Tris-HCl, pH = 6.5, 0.5% ZnCl2, 0.5% zinc acetate, 0.05% CaCl2, final pH = 6.3 79) in at least 10×volume at 4 °C for 2 h. Tissues were washed 3 times in PBS (20 min/wash). Fixed and washed tissue was dissociated by Medimax machine with Medicons-P disposable disaggregator with about 50-100 µm separator screen for 10 sec in 1 ml of PBS. Suspension was filtered through SmartStrainers 70 µm filters. Filtered cells were spun at 250 g for 3 min in a swinging bucket centrifuge at 4 °C. Supernatant was carefully removed, the cells were resuspended in 200 µL of PBS, and stored at 4 °C until sorting.
Instrument	FACSAria SORP (BD Biosciences)
Software	BD FACS DIVA software.
Cell population abundance	Sorting was conducted in Purity mode, using an 85 um nozzle with the corresponding pressure settings. The fraction of interest accounted for 2.3%-4.3% of the total cell count.
Gating strategy	Gates were set on two populations of interest: the nuclei-containing cytoplasmic parts (Hoechst33342-positive) and the YFP-containing cytoplasmic parts without nuclei (Hoechst33342-negative, YFP-bright).

☒ Tick this box to confirm that a figure exemplifying the gating strategy is provided in the Supplementary Information.
